# *N*-(4-Nitro­phen­yl)cinnamamide

**DOI:** 10.1107/S1600536809030049

**Published:** 2009-08-08

**Authors:** Aamer Saeed, Rasheed Ahmad Khera, Muhammad Shahid, Masood Parvez

**Affiliations:** a Department of Chemistry, Quaid-i-Azam University, Islamabad 45320, Pakistan; b Hamdard Institute of Pharmaceutical Sciences, Hamdard University, Islamabad Campus, Pakistan; c Department of Chemistry, The University of Calgary, 2500 University Drive NW, Calgary, Alberta, Canada T2N 1N4

## Abstract

In the mol­ecule of the title compound, C_15_H_12_N_2_O_3_, the dihedral angle between the rings is 3.04 (8)°. The central NOC_3_ fragment is planar [maximum deviation = 0.005 (3) Å] and is oriented at dihedral angles of 8.23 (8) and 7.29 (9)° with respect to the phenyl and nitro­phenyl rings, respectively. In the crystal structure, inter­molecular N—H⋯O and C—H⋯O inter­actions link the mol­ecules into a two-dimensional network. π–π contacts between rings [centroid–centroid distance = 3.719 (1) Å] may further stabilize the structure.

## Related literature

For general background to *N*-substituted benzamides, see: Beccalli *et al.* (2005 ▶); Calderone *et al.* (2006 ▶); Lindgren *et al.* (2001 ▶); Olsson *et al.* (2002 ▶); Vega-Noverola *et al.* (1989 ▶); Zhichkin *et al.* (2007 ▶). For related structures, see: Nissa *et al.* (2002 ▶, 2004 ▶); Peeters *et al.* (1986 ▶). For a description of the Cambridge Structural Database, see: Allen (2002 ▶).
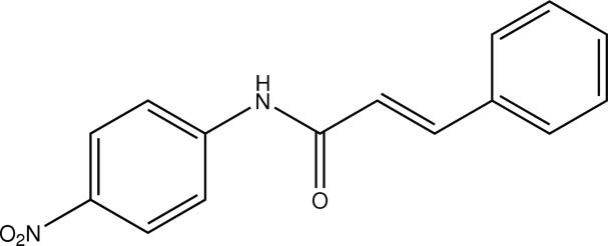


## Experimental

### 

#### Crystal data

C_15_H_12_N_2_O_3_
*M* *_r_* = 268.27
Monoclinic, 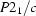

*a* = 5.903 (3) Å
*b* = 15.050 (9) Å
*c* = 14.388 (9) Å
β = 95.38 (3)°
*V* = 1272.6 (13) Å^3^
*Z* = 4
Mo *K*α radiation
μ = 0.10 mm^−1^
*T* = 173 K
0.20 × 0.18 × 0.16 mm

#### Data collection

Bruker APEXII CCD area-detector diffractometer
Absorption correction: multi-scan (*SORTAV*; Blessing, 1997 ▶) *T*_min_ = 0.980, *T*_max_ = 0.984
10408 measured reflections
2886 independent reflections
1994 reflections with *I* > 2σ(*I*)
*R*_int_ = 0.051

#### Refinement

*R*[*F* ^2^ > 2σ(*F* ^2^)] = 0.043
*wR*(*F* ^2^) = 0.117
*S* = 1.07
2886 reflections
181 parameters
H-atom parameters constrained
Δρ_max_ = 0.17 e Å^−3^
Δρ_min_ = −0.20 e Å^−3^

### 

Data collection: *COLLECT* (Hooft, 1998 ▶); cell refinement: *DENZO* (Otwinowski & Minor, 1997 ▶); data reduction: *SCALEPACK* (Otwinowski & Minor, 1997 ▶); program(s) used to solve structure: *SHELXS97* (Sheldrick, 2008 ▶); program(s) used to refine structure: *SHELXL97* (Sheldrick, 2008 ▶); molecular graphics: *ORTEP-3 for Windows* (Farrugia, 1997 ▶) and *PLATON* (Spek, 2009 ▶); software used to prepare material for publication: *SHELXTL* (Sheldrick, 2008 ▶) and *PLATON* (Spek, 2009 ▶).

## Supplementary Material

Crystal structure: contains datablocks global, I. DOI: 10.1107/S1600536809030049/hk2746sup1.cif

Structure factors: contains datablocks I. DOI: 10.1107/S1600536809030049/hk2746Isup2.hkl

Additional supplementary materials:  crystallographic information; 3D view; checkCIF report

## Figures and Tables

**Table 1:** Hydrogen-bond geometry (Å, °)

*D*—H⋯*A*	*D*—H	H⋯*A*	*D*⋯*A*	*D*—H⋯*A*
N1—H1⋯O3^i^	0.88	2.13	2.991 (2)	166
C5—H5⋯O2^ii^	0.95	2.58	3.519 (2)	168

Symmetry codes: (i) 
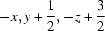
; (ii) 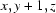.

**Fig. 1.**
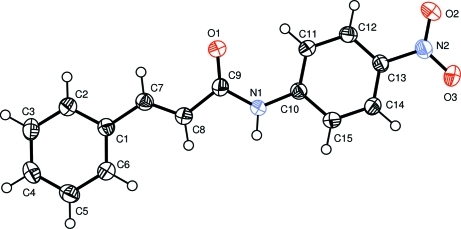
The molecular structure of the title molecule with the atom-numbering scheme. Displacement ellipsoids are drawn at the 50% probability level

**Fig. 2.**
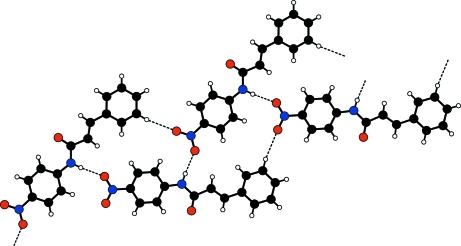
A partial packing diagram. Hydrogen bonds are shown as dashed lines.

## supplementary crystallographic information

### Comment

N-substituted benzamides, *e.g.*, declopramideare, are well known anticancer compounds and the mechanism of benzamide-induced apoptosis has been studied, (Olsson *et al.*, 2002). N-substituted benzamides inhibit the activity of nuclear factor-B and nuclear factor of activated T cells (Lindgren *et al.*, 2001). Various N-substituted benzamides exhibit potent antiemetic activity (Vega-Noverola *et al.*, 1989), while heterocyclic benzanilide are potassium channel activators (Calderone *et al.*, 2006). *N*-Alkylated 2-nitrobenzamides are intermediates in the synthesis of dibenzo[b,e][1,4]diazepines (Zhichkin *et al.*, 2007) and *N*-Acyl-2-nitrobenzamides are precursors of 2,3-disubstitued 3*H*-quinazoline-4-ones (Beccalli *et al.*, 2005). As part of our work on the structure of benzanilides and related compounds, we report herein the crystal structure of the title compound.

A search of the Cambridge Crystallographic Database (CSD version 5.30; Allen, 2002) for a fragment containing the title compound without NO_2_ group revealed only four entries containg the basic skeleton of the title compound with refcodes: DIPHUF (Peeters *et al.*, 1986), EHATUC and EHAVAK (Nissa *et al.*, 2002) and FALQAL (Nissa *et al.*, 2004).

In the molecule of the title compound (Fig. 1), rings A (C1-C6) and B (C10-C15) are, of course, planar and they are oriented at a dihedral angle of A/B = 3.04 (8)°. The (O1/N1/C7-C9) moiety is planar with a maximum deviation of -0.005 (3) Å for atom C8 and it is oriented with respect to rings A and B at dihedral angles of 8.23 (8) and 7.29 (9) °, respectively.

In the crystal structure, intermolecular N-H···O and C-H···O interactions (Table 1) link the molecules into a two dimensional network (Fig. 2), in which they may be effective in the stabilization of the structure. The π–π contact between the phenyl rings, Cg1—Cg2^i^ [symmetry code: (i) 1 - x, y - 1/2, 1/2 - z, where Cg1 and Cg2 are centroids of the rings A (C1-C6) and B (C10-C15), respectively] may further stabilize the structure, with centroid-centroid distance of 3.719 (1) Å.

### Experimental

For the preparation of the title compound, cinnamic acid was converted into cinnamoyl chloride using the standard procedure. A stirred solution of cinnamoyl chloride (5.4 mmol) in CHCl_3_ was treated with *p*-nitroaniline (21.6 mmol) under a nitrogen atmosphere at reflux for 4 h. Upon cooling, the reaction mixture was diluted with CHCl_3_ and washed consecutively with aq 1.0 *M* HCl and saturated aq NaHCO_3_. The organic layer was dried over anhydrous magnesium sulfate and concentrated under reduced pressure. Crystallization of the residue in MeOH afforded the title compound (yield; 81%) as colorless crystals: Anal. calcd. for C_15_H_12_N_2_O_3_: C, 67.16; H, 4.51; N, 10.44; found: C, 67.21; H, 4.59; N, 10.41.

### Refinement

H atoms were positioned geometrically with N-H = 0.88 Å (for NH) and C-H = 0.95 Å for aromatic H atoms, respectively, and constrained to ride on their parent atoms, with U_iso_(H) = 1.2U_eq_(C,N).

### Crystal data

**Table:**

C_15_H_12_N_2_O_3_	*F*(000) = 560
*M**_r_* = 268.27	*D*_x_ = 1.400 Mg m^−^^3^
Monoclinic, *P*2_1_/*c*	Mo *K*α radiation, λ = 0.71073 Å
Hall symbol: -P 2ybc	Cell parameters from 10408 reflections
*a* = 5.903 (3) Å	θ = 3.1–27.4°
*b* = 15.050 (9) Å	µ = 0.10 mm^−^^1^
*c* = 14.388 (9) Å	*T* = 173 K
β = 95.38 (3)°	Block, colorless
*V* = 1272.6 (13) Å^3^	0.20 × 0.18 × 0.16 mm
*Z* = 4	

### Data collection

**Table:**

Bruker APEXII CCD area-detector diffractometer	2886 independent reflections
Radiation source: fine-focus sealed tube	1994 reflections with *I* > 2σ(*I*)
graphite	*R*_int_ = 0.051
φ and ω scans	θ_max_ = 27.4°, θ_min_ = 3.1°
Absorption correction: multi-scan (*SORTAV*; Blessing, 1997)	*h* = −7→7
*T*_min_ = 0.980, *T*_max_ = 0.984	*k* = −18→19
10408 measured reflections	*l* = −18→18

### Refinement

**Table:**

Refinement on *F*^2^	Primary atom site location: structure-invariant direct methods
Least-squares matrix: full	Secondary atom site location: difference Fourier map
*R*[*F*^2^ > 2σ(*F*^2^)] = 0.043	Hydrogen site location: inferred from neighbouring sites
*wR*(*F*^2^) = 0.117	H-atom parameters constrained
*S* = 1.07	*w* = 1/[σ^2^(*F*_o_^2^) + (0.0545*P*)^2^ + 0.2254*P*] where *P* = (*F*_o_^2^ + 2*F*_c_^2^)/3
2886 reflections	(Δ/σ)_max_ < 0.001
181 parameters	Δρ_max_ = 0.17 e Å^−^^3^
0 restraints	Δρ_min_ = −0.20 e Å^−^^3^

### Special details

**Table:**

Geometry. All e.s.d.'s (except the e.s.d. in the dihedral angle between two l.s. planes) are estimated using the full covariance matrix. The cell e.s.d.'s are taken into account individually in the estimation of e.s.d.'s in distances, angles and torsion angles; correlations between e.s.d.'s in cell parameters are only used when they are defined by crystal symmetry. An approximate (isotropic) treatment of cell e.s.d.'s is used for estimating e.s.d.'s involving l.s. planes.
Refinement. Refinement of *F*^2^ against ALL reflections. The weighted *R*-factor *wR* and goodness of fit *S* are based on *F*^2^, conventional *R*-factors *R* are based on *F*, with *F* set to zero for negative *F*^2^. The threshold expression of *F*^2^ > σ(*F*^2^) is used only for calculating *R*-factors(gt) *etc*. and is not relevant to the choice of reflections for refinement. *R*-factors based on *F*^2^ are statistically about twice as large as those based on *F*, and *R*- factors based on ALL data will be even larger.

### Fractional atomic coordinates and isotropic or equivalent isotropic displacement parameters (Å^2^)

**Table:**

	*x*	*y*	*z*	*U*_iso_*/*U*_eq_	
O1	0.73930 (18)	0.43225 (7)	0.92756 (8)	0.0419 (3)	
O2	0.2344 (2)	0.02701 (7)	0.81198 (8)	0.0477 (3)	
O3	−0.0962 (2)	0.07667 (8)	0.75877 (9)	0.0534 (4)	
N1	0.3863 (2)	0.44089 (8)	0.84727 (9)	0.0327 (3)	
H1	0.2850	0.4794	0.8233	0.039*	
N2	0.1031 (2)	0.08866 (8)	0.79176 (9)	0.0368 (3)	
C1	0.7780 (2)	0.71883 (10)	0.93224 (10)	0.0299 (3)	
C2	0.9858 (3)	0.75660 (11)	0.96491 (10)	0.0355 (4)	
H2	1.1095	0.7191	0.9862	0.043*	
C3	1.0150 (3)	0.84803 (11)	0.96685 (11)	0.0416 (4)	
H3	1.1580	0.8727	0.9892	0.050*	
C4	0.8371 (3)	0.90315 (11)	0.93650 (11)	0.0415 (4)	
H4	0.8574	0.9658	0.9375	0.050*	
C5	0.6279 (3)	0.86698 (11)	0.90433 (11)	0.0401 (4)	
H5	0.5047	0.9048	0.8835	0.048*	
C6	0.5991 (3)	0.77560 (10)	0.90259 (10)	0.0346 (4)	
H6	0.4553	0.7513	0.8809	0.041*	
C7	0.7580 (2)	0.62167 (10)	0.92964 (10)	0.0316 (4)	
H7	0.8827	0.5887	0.9584	0.038*	
C8	0.5822 (2)	0.57539 (10)	0.89084 (10)	0.0334 (4)	
H8	0.4535	0.6061	0.8621	0.040*	
C9	0.5825 (2)	0.47700 (10)	0.89143 (10)	0.0316 (4)	
C10	0.3266 (2)	0.35165 (10)	0.83539 (10)	0.0282 (3)	
C11	0.4746 (2)	0.28151 (10)	0.85797 (10)	0.0316 (4)	
H11	0.6261	0.2930	0.8834	0.038*	
C12	0.4016 (3)	0.19496 (10)	0.84341 (10)	0.0322 (4)	
H12	0.5021	0.1467	0.8586	0.039*	
C13	0.1807 (2)	0.17939 (9)	0.80651 (10)	0.0300 (3)	
C14	0.0315 (2)	0.24827 (10)	0.78241 (10)	0.0323 (4)	
H14	−0.1191	0.2362	0.7562	0.039*	
C15	0.1037 (2)	0.33422 (10)	0.79678 (10)	0.0315 (3)	
H15	0.0026	0.3821	0.7806	0.038*	

### Atomic displacement parameters (Å^2^)

**Table:**

	*U*^11^	*U*^22^	*U*^33^	*U*^12^	*U*^13^	*U*^23^
O1	0.0340 (6)	0.0328 (6)	0.0563 (7)	0.0019 (5)	−0.0103 (5)	−0.0006 (5)
O2	0.0508 (7)	0.0283 (6)	0.0626 (8)	0.0039 (6)	−0.0020 (6)	0.0050 (5)
O3	0.0390 (7)	0.0386 (7)	0.0794 (9)	−0.0086 (5)	−0.0118 (6)	−0.0074 (6)
N1	0.0305 (7)	0.0242 (7)	0.0418 (7)	0.0007 (5)	−0.0051 (5)	0.0016 (6)
N2	0.0403 (8)	0.0300 (7)	0.0397 (8)	−0.0026 (6)	0.0021 (6)	−0.0015 (6)
C1	0.0325 (8)	0.0302 (8)	0.0269 (7)	−0.0008 (6)	0.0027 (6)	−0.0009 (6)
C2	0.0356 (8)	0.0348 (9)	0.0354 (8)	−0.0007 (7)	−0.0012 (6)	−0.0009 (7)
C3	0.0430 (10)	0.0373 (9)	0.0436 (10)	−0.0102 (8)	−0.0009 (7)	−0.0050 (7)
C4	0.0543 (10)	0.0278 (9)	0.0426 (10)	−0.0049 (8)	0.0057 (8)	−0.0017 (7)
C5	0.0453 (10)	0.0328 (9)	0.0420 (9)	0.0059 (8)	0.0024 (7)	0.0025 (7)
C6	0.0328 (8)	0.0346 (9)	0.0358 (8)	−0.0008 (7)	0.0009 (6)	−0.0010 (7)
C7	0.0330 (8)	0.0311 (8)	0.0307 (8)	0.0010 (7)	0.0027 (6)	0.0005 (6)
C8	0.0318 (8)	0.0306 (8)	0.0370 (8)	−0.0003 (7)	−0.0003 (6)	0.0021 (7)
C9	0.0307 (8)	0.0303 (8)	0.0337 (8)	−0.0026 (7)	0.0016 (6)	0.0003 (6)
C10	0.0298 (8)	0.0272 (8)	0.0275 (7)	0.0004 (6)	0.0019 (6)	−0.0002 (6)
C11	0.0282 (8)	0.0311 (8)	0.0344 (8)	0.0008 (6)	−0.0029 (6)	−0.0004 (6)
C12	0.0330 (8)	0.0298 (8)	0.0332 (8)	0.0036 (7)	−0.0009 (6)	0.0001 (6)
C13	0.0329 (8)	0.0248 (7)	0.0322 (8)	−0.0016 (6)	0.0027 (6)	−0.0007 (6)
C14	0.0271 (7)	0.0332 (8)	0.0363 (8)	−0.0016 (7)	0.0013 (6)	−0.0020 (7)
C15	0.0283 (7)	0.0314 (8)	0.0343 (8)	0.0033 (6)	0.0010 (6)	0.0016 (7)

### Geometric parameters (Å, °)

**Table:**

O1—C9	1.2207 (18)	C5—H5	0.9500
O2—N2	1.2264 (17)	C6—H6	0.9500
O3—N2	1.2397 (17)	C7—C8	1.329 (2)
N1—C9	1.3790 (19)	C7—H7	0.9500
N1—C10	1.395 (2)	C8—C9	1.481 (2)
N1—H1	0.8800	C8—H8	0.9500
N2—C13	1.450 (2)	C10—C11	1.389 (2)
C1—C2	1.393 (2)	C10—C15	1.404 (2)
C1—C6	1.394 (2)	C11—C12	1.382 (2)
C1—C7	1.467 (2)	C11—H11	0.9500
C2—C3	1.387 (2)	C12—C13	1.381 (2)
C2—H2	0.9500	C12—H12	0.9500
C3—C4	1.377 (2)	C13—C14	1.383 (2)
C3—H3	0.9500	C14—C15	1.372 (2)
C4—C5	1.389 (2)	C14—H14	0.9500
C4—H4	0.9500	C15—H15	0.9500
C5—C6	1.386 (2)		
			
C9—N1—C10	128.87 (13)	C1—C7—H7	116.9
C9—N1—H1	115.6	C7—C8—C9	121.43 (14)
C10—N1—H1	115.6	C7—C8—H8	119.3
O2—N2—O3	122.46 (13)	C9—C8—H8	119.3
O2—N2—C13	119.59 (13)	O1—C9—N1	123.31 (14)
O3—N2—C13	117.95 (13)	O1—C9—C8	123.67 (14)
C2—C1—C6	118.10 (14)	N1—C9—C8	113.01 (13)
C2—C1—C7	118.78 (14)	C11—C10—N1	123.82 (13)
C6—C1—C7	123.12 (13)	C11—C10—C15	119.74 (14)
C3—C2—C1	121.03 (15)	N1—C10—C15	116.44 (13)
C3—C2—H2	119.5	C12—C11—C10	120.02 (14)
C1—C2—H2	119.5	C12—C11—H11	120.0
C4—C3—C2	120.12 (15)	C10—C11—H11	120.0
C4—C3—H3	119.9	C13—C12—C11	119.22 (14)
C2—C3—H3	119.9	C13—C12—H12	120.4
C3—C4—C5	119.84 (15)	C11—C12—H12	120.4
C3—C4—H4	120.1	C12—C13—C14	121.69 (14)
C5—C4—H4	120.1	C12—C13—N2	119.36 (13)
C6—C5—C4	119.92 (15)	C14—C13—N2	118.95 (14)
C6—C5—H5	120.0	C15—C14—C13	119.18 (14)
C4—C5—H5	120.0	C15—C14—H14	120.4
C5—C6—C1	120.97 (15)	C13—C14—H14	120.4
C5—C6—H6	119.5	C14—C15—C10	120.14 (14)
C1—C6—H6	119.5	C14—C15—H15	119.9
C8—C7—C1	126.22 (14)	C10—C15—H15	119.9
C8—C7—H7	116.9		
			
C6—C1—C2—C3	−0.8 (2)	C9—N1—C10—C15	172.82 (14)
C7—C1—C2—C3	178.57 (14)	N1—C10—C11—C12	−179.62 (14)
C1—C2—C3—C4	0.2 (2)	C15—C10—C11—C12	−0.7 (2)
C2—C3—C4—C5	0.3 (2)	C10—C11—C12—C13	−0.1 (2)
C3—C4—C5—C6	−0.2 (2)	C11—C12—C13—C14	1.0 (2)
C4—C5—C6—C1	−0.4 (2)	C11—C12—C13—N2	−179.58 (14)
C2—C1—C6—C5	0.9 (2)	O2—N2—C13—C12	−0.2 (2)
C7—C1—C6—C5	−178.44 (14)	O3—N2—C13—C12	179.82 (14)
C2—C1—C7—C8	−171.67 (14)	O2—N2—C13—C14	179.28 (14)
C6—C1—C7—C8	7.6 (2)	O3—N2—C13—C14	−0.7 (2)
C1—C7—C8—C9	179.17 (14)	C12—C13—C14—C15	−1.0 (2)
C10—N1—C9—O1	0.5 (2)	N2—C13—C14—C15	179.59 (13)
C10—N1—C9—C8	−179.20 (14)	C13—C14—C15—C10	0.1 (2)
C7—C8—C9—O1	0.8 (2)	C11—C10—C15—C14	0.8 (2)
C7—C8—C9—N1	−179.53 (14)	N1—C10—C15—C14	179.72 (13)
C9—N1—C10—C11	−8.3 (2)		

### Hydrogen-bond geometry (Å, °)

**Table:**

*D*—H···*A*	*D*—H	H···*A*	*D*···*A*	*D*—H···*A*
N1—H1···O3^i^	0.88	2.13	2.991 (2)	166
C5—H5···O2^ii^	0.95	2.58	3.519 (2)	168

Symmetry codes: (i) −*x*, *y*+1/2, −*z*+3/2; (ii) *x*, *y*+1, *z*.
